# Remote Measurement of the Angular Velocity Vector Based on Vectorial Doppler Effect Using Air-Core Optical Fiber

**DOI:** 10.34133/2022/9839502

**Published:** 2022-09-02

**Authors:** Zhenyu Wan, Yize Liang, Xi Zhang, Ziyi Tang, Liang Fang, Zelin Ma, Siddharth Ramachandran, Jian Wang

**Affiliations:** ^1^Wuhan National Laboratory for Optoelectronics and School of Optical and Electronic Information, Huazhong University of Science and Technology, Wuhan, 430074 Hubei, China; ^2^Optics Valley Laboratory, Wuhan, 430074 Hubei, China; ^3^Boston University, 8 St. Mary's St., Boston 02215, USA

## Abstract

Rotational Doppler effect has made tremendous development in both theoretical and applied research over the last decade. Different from the inertial thinking of focusing on the scalar field dominated by helical phase light, we have revealed a vectorial Doppler effect in our previous work, which is based on the spatially variant polarized light fields to simultaneously acquire the speed and direction of a target. Here, further, we propose a method to construct a flexible and robust velocimeter based on that novel effect by employing an air-core fiber with kilometer-length scale for remotely measuring the vectorial information of angular velocity in situ. In addition, we experimentally substantiate that the measurement system still has commendable accuracy in determining the direction of movement even when the air-core fiber is interfered by the external environment. The demonstrations prove the potential of vectorial Doppler effect in practical scenarios and remote measurements.

## 1. Introduction

The laser Doppler effect is an indispensable optical method for direct measurement of velocity, on account of having advantages such as high-spatial resolution, wide measurement range, and noncontact characteristics [1–4]. Based on the laser Doppler effect, one can calculate the speed of a moving object by detecting the frequency shift of the light reflected from the moving object. Particularly, a traditional case, known as the linear Doppler effect, focuses on the relationship between Doppler shift and longitudinally translational velocity [5–7]. With the exploration of helical phase light, another basic form of motion, i.e., the lateral rotation, has been connected to Doppler shift with the orbital angular momentum (OAM) as a bridge, which is called the rotational Doppler effect and has been widely studied [8–16]. Since the angular velocity vector is directional, light in this effect will be subjected to a degree of blue or red shift according to the two directions of relative motion. However, due to the ultrahigh inherent frequency of the light wave, it is not practical to directly detect the blue or red shift of light around the inherent frequency by sensing the fluctuation of light intensity. Generally, the extraction of rotational Doppler shift of light is usually realized by two detection techniques [17], such as interfering with coherent reference light [18–23] or by means of the Doppler difference between different phase vortex modes [24–28], either of which also results in only obtaining the absolute value of the frequency shift.

Evidently, the electric field of light is a vector quantity, which contains both magnitude and direction, and therefore, the vector nature of the light itself is extremely important on many occasions. Among different types of light beams, the helical phase light field, whose phase pattern of the oscillation amplitude possesses a transverse spatial structure, is considered a typical scalar beam [29–34]. In contrast, the vector state of the light field means that its polarization pattern is not inhomogeneously distributed transversely, and it is also termed as a vectorial polarization field [35–39]. In recent years, such light fields have been extensively studied and rapidly developed in multifarious applications such as super-resolution imaging, laser machining, quantum information, optical tracking, and optical sensing [40–46]. In our previous work, we revealed the concept of a vectorial Doppler effect with the spatially variant polarized light fields. Based on this novel effect, we experimentally achieved a simultaneous acquisition of the rotational speed and direction of the target by utilizing the cylindrical vector beam without any dynamic optical modulation components [47]. Nevertheless, such a system is laborious to be adopted in scenes outside the laboratory and to measure a remotely rotating target, because the generation of vectorial polarization fields in free space requires precise alignment and the system is too bulky. Fortunately, there is a medium that is very friendly to the transmission of cylindrical vector beam, known as the air-core optical fiber [48]. Different from conventional weakly guiding optical fibers, the air-core fiber can support higher-order vector beams transmission. In addition, even under the interference of the environment, most of the crosstalk of the transmitted vector beams only occurs between even and odd modes in the same mode group in the air-core fiber.

Here, we propose and experimentally demonstrate an approach to construct a flexible and robust velocimeter for remotely measuring the vectorial information of angular velocity in situ. An air-core fiber with kilometer-length scale is employed to investigate the potential of vectorial Doppler effect in actual remote measurement. Through this medium, the probe optical field is transmitted to the remote end for experimental detection of a rotating target. The results show that a clearly distinguishable peak in the spectrum of each detected signal can be observed at a frequency matching to the magnitude of angular velocity, and the directions of different angular velocities can be readily distinguished according to the dissimilitude in phase spectra of varied detection signals. Furthermore, the performance of the velocimeter is tested considering the perturbation of air-core fiber. The experimental results are extremely in line with expectations when the fiber is static, in-plane bent by stressing, or after previous perturbations. It is observed that the geometric phase induced by out-plane movement of air-core fiber results in fluctuations of the Doppler shift. Significantly, we show that the measurement system has commendable accuracy in determining the direction of movement even when it is interfered by the external environment. In a sense, this presented method might be a promising candidate for facilitating applications of vectorial Doppler effect in remote sensing and in vivo measurement.

## 2. Results

### 2.1. Concept and Principle

In the air-core fiber, the feature of air-core enables conservation of OAM states, and both the HE_*ℓ*+1,1_ and EH_*ℓ*−1,1_ vector modes exist as a coherent superposition of the OAM modes. The integer *ℓ* refers to the absolute value of the topological charge of the OAM modes used for linear combination to obtain the vector modes. Concretely speaking, the states of polarization of cylindrical vector beams are a linear combination of orthogonal circularly polarized optical vortices with opposite topological charges. As well known, the optical vortices can carry high-dimensional OAM related to the helical wavefront described by the azimuthal phase factor exp(*iℓϕ*), where *ϕ* is the azimuthal coordinate. By derivation, for monochromatic paraxial light, the electric field of the vector eigenmode in the air-core fiber can be described using Jones vector as

(1) $$\begin{array}{c} E\left(r,\phi \right)=A_{0}\left(r\right)\cdot \left[\begin{array}{c} \cos\left(\ell \phi -\alpha \right)\\ -\sigma \cdot \sin\left(\ell \phi -\alpha \right) \end{array}\right], \end{array}$$

where *A*_0_(*r*) is the real amplitude of electric field and *σ* takes two values of +1 and −1 corresponding to the two types of cylindrical vector fields analogous to HE_*ℓ*+1,1_ and EH_*ℓ*−1,1_ vector modes, respectively. Note that the phase factor *α* here is derived from the initial phase difference between the two OAM components of the synthesized vector mode and determines the initial polarization orientation of the vector mode. When the geometric perturbation of the air-core fiber occurs, the OAM modes will obtain a so-called Pancharatnam phase without coupling to other orders, and the vector modes will also remain in the original mode group due to the interchangeability between the vector and phase vortex modes [49]. Besides, most of the crosstalk of the vector modes only occurs in the same mode group, especially only existing in mixing between the odd-even degenerate counterparts. Remarkably, this intracrosstalk corresponds to a change of phase factor *α*, thereby resulting in the intuitive rotation of the transverse polarization distribution of synthesized vector beam (see Supplementary Materials (available here)).

**Figure 1:**
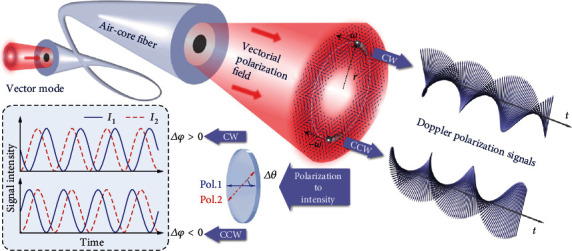
Concept and schematic of measuring the angular velocity vector using air-core fiber. Through the excitation of the vector eigenmode by the coupling of the vector mode, the air-core fiber robustly launches a vectorial polarization field, which has the vectorial characteristic featuring spatial polarization. By illuminating the object with normally incident probe light, they will reflect the Doppler polarization signal light with time-varying polarization. Then, the Doppler polarization signal is projected into two different directions of polarizing angle to determine the rate and orientation of its varying polarization. Two time-varying intensity signals corresponding to *θ*_1_- and *θ*_2_-polarized delay/advance for distinguishing the chirality of the Doppler polarization signal.

Figure 1 shows the concept and schematic of measuring the angular velocity vector by using air-core optical fiber. Generally, based on a scalar optical field, such as phase-twisted beam, or lateral intensity-varied beam with superposing opposite charges, only the magnitude of Doppler shift can be extracted from the detected Doppler time-varying signal. Consequently, one can only derive the magnitude of the angular velocity without distinguishing the direction of movement. Here, through the excitation of the vector eigenmode, the air-core fiber robustly propagates a vectorial polarization field. The fiber-guided vectorial polarization field which has the vectorial characteristic featuring spatial polarization is utilized to measure the angular velocity vector **Ω** of a small object, such as a microparticle. By illuminating the object with normally incident probe light, meaning that the beam axis of the probe light is consistent with the rotation axis of the object, there will be a back-reflected signal light with time-varying polarization that depends on the spatial location of the scatterer. According to our previous comprehension, the reflected signal light can be interpreted as a Doppler polarization signal (DPS), expressed as

(2) $$\begin{array}{c} E\left(\Omega ,t\right)=A\cdot \left[\begin{array}{c} \cos\left(\ell \Omega t-\alpha \right)\\ -\sigma \cdot \sin\left(\ell \Omega t-\alpha \right) \end{array}\right], \end{array}$$

where *A* is the real amplitude of the signal light and *Ω* takes the plus or minus signs corresponding to the movement of counterclockwise (CCW) or clockwise (CW) directions, respectively. The DPS, characterized by the polarization varying over time, is essential for revealing the vectorial Doppler effect, in which the rate of polarization change is mapped to the magnitude of the Doppler shift, and the handedness of polarization change corresponds to its sign. This means that when the rotational direction of the target is reversed, the DPSs would show different chirality, which can be used to distinguish the direction of the angular velocity.

In practice, the change of linear polarization can be roughly identified by its projection in a fixed direction, and more accurately here, we project the DPS into two different directions to determine the rate and orientation of its varying polarization. According to this, the intensity of the DPS after passing through the polarizer can be derived as

(3) $$\begin{array}{c} I_{j}\left(\Omega ,t,\theta \right)={\left|\left(\begin{array}{cc} \cos^{2}\theta_{j} & \cos\theta_{j}\sin\theta_{j}\\ \cos\theta_{j}\sin\theta_{j} & \sin^{2}\theta_{j} \end{array}\right)\cdot E\left(\Omega ,t\right)\right|}^{2}=i_{0}\left\{1+\cos\left[2\left(\ell \Omega t+{\sigma \theta }_{j}-\alpha \right)\right]\right\}, \end{array}$$

where *θ*_*j*_ is the polarizing-angle of the polarizer with respect to the *x-*axis, and *j* specifically takes the value of 1 and 2, referring to the two directions of the polarizers as shown in Figure 1 (Pol.1, Pol.2). It shows that there are two valuable pieces of information in the intensity signal: one is the frequency term related to the rotational speed, and the other is the phase term related to the polarizing angle and the geometric perturbation of the air-core fiber during the transmission of the vector mode.

Through frequency spectrum analysis, we can extract a uniform Doppler shift from both *θ*_1_- and *θ*_2_-polarized intensity components of the DPS expressed as

(4) $$\begin{array}{c} \left|\Delta f\right|=\frac{1}{2\pi }\left|2\ell \Omega \right|. \end{array}$$

Obviously, this is the situation where the fiber is stationary during the measurement. When the fiber is geometrically perturbed, the frequency shift value may fluctuate slightly, which depends on the partial derivative of the phase factor *α* with time.

In addition to the acquirement of the frequency shift value from Fourier spectrum analysis, the Fourier transform can also obtain the phase spectrum, which is an extremely helpful tool for picking up the sign of the frequency shift. Typically, there is a lagging or leading relationship between the two time-varying intensity signals corresponding to *θ*_1_- and *θ*_2_-polarized. It gives a specific relative phase difference at the frequency peaks in the Fourier phase spectra between the two polarization components, written as

(5) $$\begin{array}{c} \Delta \varphi =\varphi_{2}-\varphi_{1}=\mathrm{sign}\left(\Omega \right)\cdot 2\sigma \Delta \theta , \end{array}$$

where sign(*Ω*) represents the sign of angular velocity *Ω* and Δ*θ* is the deviation of two polarizing-angles. Note that the relative phase difference will not be affected too much by the geometric perturbation of air-core fiber due to the fact that the phase factor *α* caused by the geometric perturbation simultaneously exists in the two signal components with the same value. It is apparent that the sign of the relative phase difference has a clear correspondence with the direction of angular velocity, which also means that the chirality of DPS has been effectively distinguished.

Hence, the full vector information of angular velocity can be measured at once by performing Fourier analysis on the two polarization components decomposed by the DPS, in which the extracted relative phase difference can be regarded as a new Doppler phase shift, as an important supplementary of the Doppler frequency shift. These unified Doppler frequency shift and Doppler phase shift, which play a key role in vectorial Doppler metrology, break the technical restriction of traditional laser Doppler metrology based on scalar light fields. It is remarkable that the Doppler shift and relative phase difference in the vectorial Doppler effect are wavelength independent, as given in Equations (4) and (5), enabling the velocimeter to naturally against the spectral broadening or sideband increase caused by the fiber nonlinear effects. In addition, the use of air-core fiber, far from disrupting the judgment of rotational direction, brings several distinct advantages to the measurement system, such as flexibility and promise for remote sensing. Although the geometric perturbation of the air-core fiber may cause the fluctuation of Doppler shift, if the perturbation is random, then the influence can be eliminated by averaging through multiple measurements. In a sense, such a system would also be quite robust.

### 2.2. Experimental Realization

**Figure 2:**
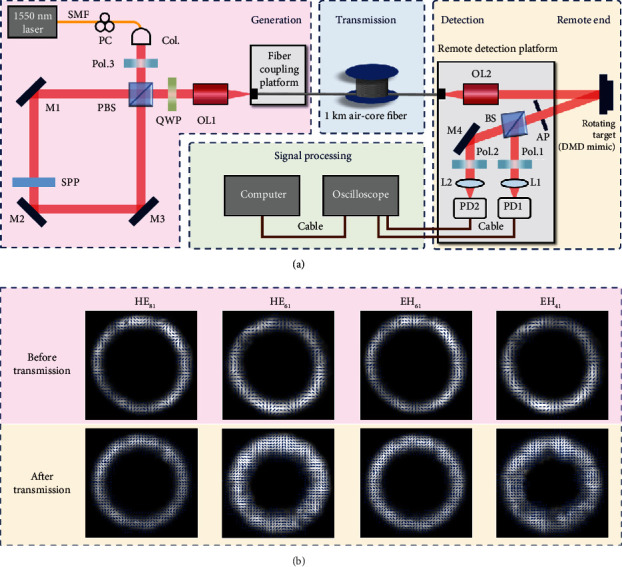
Experimental setup for the measurement of angular velocity vector remotely and robustly. (a) In the generation part, the Sagnac interferometer configuration is constructed to produce the stable and high-quality cylindrical vectorial polarization fields. In the transmission part, the vector light is coupled into a 1 km air-core fiber for transmission to the remote end (perturbation is to simulate the realistic test scenario). In the detection part, the emission of probe light and the collection of signal light are integrated on one platform for the detection of remote rotating target. In the signal processing part, Fourier analysis of the signals to extract the full angular velocity vector information; Col.: collimator; Pol.1, Pol.2, Pol.3: polarizer; PBS: polarization beam splitter; M1, M2, M3, M4: mirror; SPP: spiral phase plate; QWP: quarter-wave plate; OL1, OL2: objective lens; AP: aperture; BS: beam splitter; L1, L2: lens; PD1, PD2: photodetector; DMD: digital micromirror device. (b) The light field reconstruction records (the intensities and polarization distributions) of several typically generated vector beams before and after transmission through the air-core fiber in the experiment.

We construct an experimental setup for the remote and robust measurement of angular velocity vector, as illustrated in Figure 2(a), which can be divided into four parts: generation, transmission, detection, and signal processing. At the beginning of the generation part, a laser diode with 10 dBm at 1550 nm is employed to generate a fundamental Gaussian beam, which is then output from single-mode fiber after adjusting the polarization through a polarization controller and then collimated by a collimator. The subsequent polarizer (Pol.3) at 45° ensures that the power of light can be divided evenly in *x* and *y* polarization directions. Through a Sagnac interferometer configuration, the cylindrical vector beam is created (see Materials and Methods for details). In this part, a spiral phase plate (SPP) converts the Gaussian beam to helical phase beam, and the light beam in each path is modulated to the opposite topological charge in *x*/*y* linear polarization. After passing through a quarter-wave plate (QWP) at 45°, the superposed beam becomes a cylindrical vectorial polarization field. Subsequently, the vector light is coupled into a 1 km air-core fiber by the objective lens (OL1), thus entering the transmission part, and finally being transmitted to the remote end. Due to the loss of Sagnac configuration and coupling loss, the incoming fiber power is within 5 dBm, and hence, the nonlinear effect is weak to be ignored in the experiment. In order to realize a more realistic test scenario, we also apply perturbation caused by bending or shaking to the transmission part experimentally. The light field reconstruction records of several typically generated vector beams before and after transmission through the air-core fiber in the experiment are shown in Figure 2(b) (see Supplementary Materials for the recording method). One can see that these vector beams still maintain quite good vector characteristics after being transmitted through the air-core fiber. The quantitative assessment of this maintaining is performed, showing that the polarization purity is not less than 89% for each mode group before and after 1 km air-core fiber (see Supplementary Materials for the calculated values and methods).

Output from the air-core fiber and collimated into the free space at the remote end by OL2, the vector beam is regarded as the probe light in the detection part. The probe light illuminates on the rotating microparticle at normal incidence and ensuring that the center of the probe light coincides with the center of rotation. Here, we employ a digital micromirror device (DMD) to mimic the rotating microparticle (see Materials and Methods for details). When reflected by the rotating particle from the probe light, a string of signal light, i.e., the DPS, is selected by an aperture (AP) for Doppler metrology, containing the information of the angular velocity vector. The beam splitter (BS) is used to separate the signal light into two paths of equal power, which are, respectively, injected into the photodetectors (PD1, PD2) by lenses (L1, L2) after passing through the polarizers (Pol.1, Pol.2) in different polarization directions. Furthermore, we integrate the port that illuminates the target and the other one that receives the scattered light on one platform, so our detection device at the remote end is compact and easy-adjusting. Eventually, the signals recorded by the photodetectors are transmitted to the signal processing part by the cables. In this experiment, we utilize an oscilloscope to convert analog signals into digital form and use a computer to perform the fast Fourier transform (FFT), all of which can also be integrated into a unit module in the engineering scene. In Fourier amplitude spectrum, a Doppler peak is searched and its corresponding frequency value is recorded as the magnitude of Doppler shift |Δ*f*|. By subtracting the Fourier phase spectra of the two intensity signals, the relative phase difference Δ*φ* is recorded at the frequency |Δ*f*|. As a result, the Doppler shift and relative phase difference are obtained to calculate the full angular velocity vector information of the remotely rotating target.

### 2.3. Remote Measurement of Angular Velocity Vector

**Figure 3:**
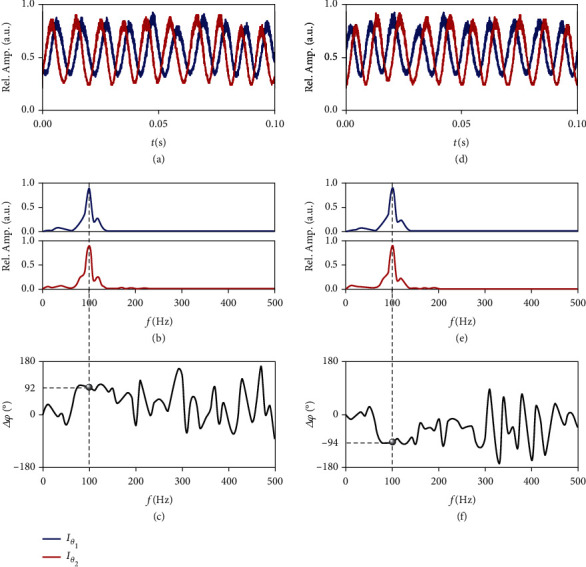
Measured signals and Fourier analysis for the rotating target based on the vectorial eigenmode HE_61_ in air-core fiber. (a–c) Measured results for counterclockwise motion with *Ω* = 20*π* rad/*s*. (d–f) Measured results for clockwise motion with *Ω* = −20*π* rad/s. (a, d) Measured Doppler intensity signals by PD1 and PD2 after filtering the DPS through two polarizers, respectively. (b, e) Calculated Fourier amplitude spectra for obtaining a Doppler shift peak. (c, f) calculated relative Fourier phase spectra between two intensity signals for obtaining the relative phase difference matching the frequency peak.

Initially, we measure the rotating target based on the vectorial eigenmode HE_61_ in air-core fiber to substantiate our method experimentally, as shown in Figure 3. In this case, the deviation of two polarizing-angles Δ*θ* (angle difference between Pol.1 and Pol.2) is 45°. As the mimicked microparticle rotating counterclockwise with *Ω* = 20*π* rad/*s*, we detect Doppler intensity signals by PD1 and PD2 after filtering the DPS through two polarizers, respectively, as plotted in Figure 3(a). By performing Fourier analysis on these intensity signals, the calculated Fourier amplitude (Figure 3(b)) and phase (Figure 3(c)) spectra are gotten for obtaining a Doppler shift peak and the matching relative phase difference. Likewise, as the mimicked microparticle rotating is reversed with *Ω* = −20*π* rad/*s*, we acquire another set of data (Figures 3(d)–3(f)). It is obvious that no matter which direction the microparticle rotates, a clear and identical frequency peak at 100 Hz appears in these amplitude spectra. From the comparison, one can clearly see that the two intensity signals show a significant lead or lag relationship in different direction of rotation, and this can be quantitatively described by the opposite relative phase difference around ±90°. Consequently, the magnitude and sign of the Doppler shift are all determined, and as a result, the angular velocity vector is conveniently extracted. In addition, the vectorial eigenmodes HE_81_ and EH_61_ are also employed to implement the same experiment, and the comparison results show that the relative phase difference at the Doppler shift peak also reversed the sign (see Supplementary Materials).

**Figure 4:**
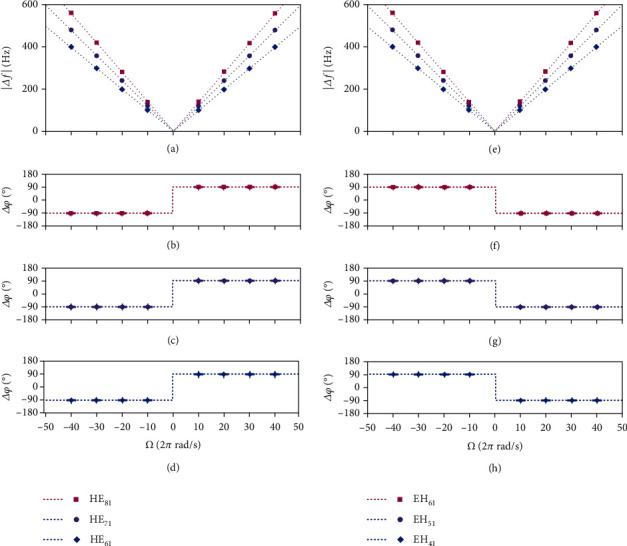
Measured results based on 6 distinct vectorial eigenmodes in the air-core fiber under different rotation states. (a, e) Measured magnitude of Doppler shifts, shown as square, round and rhombic points, respectively, and compared to the theoretical prediction, shown as dashed lines in different colors. Measured relative phase differences matching the frequency shifts via HE_81_ in (b), HE_71_ in (c), HE_61_ in (d), EH_61_ in (f), EH_51_ in (g), and EH_41_ in (h).

Furthermore, we utilize 6 distinct vectorial eigenmodes (HE_81_, HE_71_, HE_61_, EH_61_, EH_51_, and EH_41_) in the air-core fiber to get some measurement results under different states of angular velocity while keeping the fiber static, as shown in Figure 4 (each data point is obtained through 5 measurements). Among them, Figures 4(a) and 4(e) show the measured magnitude of Doppler shifts, and Figures 4(b)–4(h) are relative phase differences matched with the frequency shifts correspondingly. The results indicate that the detected Doppler shift changes linearly with the magnitude of the angular velocity and is proportional to the order of the vector mode, while the sign of the relative phase difference alters according to the direction of motion. This sign reversal observed here is a distinct feature that clearly distinguishes the vectorial Doppler effect from the conventional Doppler effect with scalar field. In addition, when the vector mode is varied from HE-liked type to EH-liked type, i.e., *σ* in Equation (5) varies from positive to negative; the regularity of the relative phase difference will be reversed, which means that the measurement system should determine the type of vector mode before being applied. These experimental results are distinctly in line with the theory expected by Equation (4) and Equation (5). Hence, it becomes possible to accurately obtain the magnitude and direction of the angular velocity by a single measurement.

### 2.4. System Performance under Fiber Perturbations

**Figure 5:**
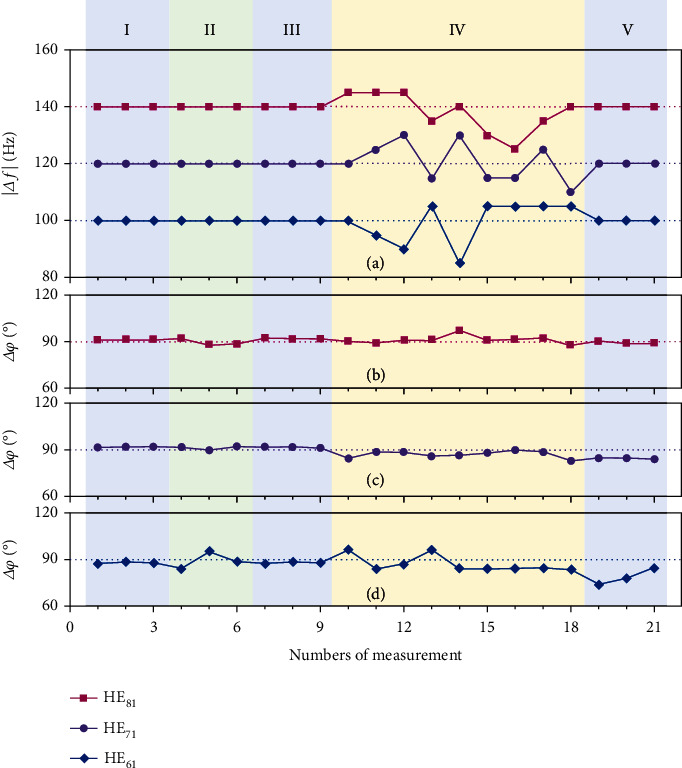
Measured results for the air-core fiber under static state and external interference. (a) Measured magnitude of Doppler shifts by multiple numbers of measurement. Measured relative phase differences matching the frequency shifts via HE_81_ in (b), HE_71_ in (c), and HE_61_ in (d). Regions I, III, and V are the cases where the air-core fiber remains static state, and region II is the case where the air-core fiber is stressed (in-plane bent), and region IV is the case where the air-core fiber is subjected to waggling (out-plane movement).

We further test the performance of our system in various scenarios by switching the state of the air-core fiber under different situations, such as static, stressed (in-plane bent), and waggling (out-plane movement), as shown in Figure 5. The results are obtained under Δ*θ* = 45° and the counterclockwise rotation with *Ω* = 20*π* rad/*s*. According to the measured magnitude of Doppler shifts (Figure 5(a)) and the relative phase differences (Figures 5(b)–5(d)) by multiple numbers of measurement, one can clearly see that the results are extremely consistent when the fiber is static, even after previous interference with the fiber. Besides, a slight press of the fiber will not distort the results, whereas a wobble of the fiber might make a more dramatic impact on the measured Doppler shift, which fluctuates with the fiber waggling. This interesting phenomenon can be attributed to whether the geometric phase will accumulate with time; in other words, whether the solid angle enclosed by the *k*-vector of the light path will change as the fiber moves. Region II in Figure 5 corresponds to an in-plane perturbation which will lead to birefringence, while region IV is an out-plane movement that will cause an extra geometric phase [49]. Since OAM eigenmodes in the air-core fiber are stable against birefringence [48], the vector mode, as a linear combination of OAM modes with opposite total angular momenta, also possesses a similar stability to in-plane perturbation. In contrast, the geometric phase induced by out-plane movement will distort this linear combination, causing the crosstalk of vector modes inside the corresponding mode group, as observed intuitively that the vector beam rotates laterally around its center (see Supplementary Materials). A more detailed theoretical analysis for this phenomenon is available in Ref. [49]. As a result, this gives rise to the fluctuation of Doppler shift. For this, by averaging through multiple measurements under the geometric perturbation, the mean frequency shift still maintains a high degree of fit with the theoretical value. Concretely, the measured mean frequency shifts based on HE_61_, HE_71_, and HE_81_ vector modes are 99.4 Hz, 120.6 Hz, and 137.8 Hz, respectively. The error between these results and the theoretical value is within 2%, and it can be further reduced by increasing the number of measurements. In addition, it can be found that a more significant characteristic of this measurement system is that the relative phase difference always keeps a similar value and its sign does not reverse at will no matter whether the fiber is static or disturbed, which makes the determination of the direction of angular velocity vector very robust. The stable observations of the relative phase difference are owing to the large effective index difference between mode groups (see Methods for details). On the other hand, the out-plane geometric perturbations fundamentally lead to mode mixing in the vector basis, or particularly only intracrosstalk within a vector mode group, but not in the OAM basis.

## 3. Discussion

For a measurement system, the precision and accuracy are typically important indicators. In our proposed velocimeter, there are several ways to obtain high-quality measured results. Firstly, we need to fine-tune the coupling of the vector modes from free space to the air-core fiber to excite purer vector modes, because the different mode groups will affect the signal-to-noise ratio. Secondly, the alignment of the probe light and the rotating target is also very crucial. The mismatch between the center of the probe light and the center of the rotation results in the stretching of the Doppler shift peak [50]. This problem can be suppressed by introducing feedback mechanism into the system and using the method of adaptive tracking adjustment. Moreover, one can also improve the resolution of Fourier amplitude and phase spectra in signal processing by increasing the time of data acquisition. On the other hand, in the case of long-range detection, however, it is required to achieve echo receiving at a long distance, which means that the received signals are to travel back from remote end before being detected and/or processed. There are two approaches of technical optimization that may provide an improvement for this work. The first potential way is to probe the echo signal light at the remote end and then backhaul the two recorded signals across two long cables for in situ processing. Furthermore, another option is to couple the echo signal light into two optical fibers after dual-polarization splitting at the remote end and then backhaul the two projected signals over two lengthy fiber channels for detection and processing in situ. The fiber channels there could be two multimode fibers or two occupied cores of a designed multicore fiber [51].

In summary, we have proposed a method to construct a flexible and robust velocimeter based on the vectorial Doppler effect by employing an air-core fiber with kilometer-length scale for remotely measuring the vectorial information of angular velocity in situ. This method relies on the excellent transmission performance of higher-order vector modes in the air-core fiber and the characteristic of spatially varying polarization distribution of the vector mode. As we have substantiated experimentally, the vector mode is transmitted to the remote end with exceedingly high quality for measurement, and even if there is an external disturbance in the transmission path, the effectiveness of the whole measurement system will not be damaged greatly. The significance of the method is that the detection port has a high degree of flexibility and the transmission channel is small as hair in cross-section and is extendable in length with low loss, enabling the potential in remote measurements and in vivo scenarios. In addition, the method takes advantage of the newly developed Doppler effect to ensure that the magnitude and direction of the angular velocity vector are obtained at once. Our method may offer significant application prospects, such as monitoring the rotation vectors in bioinstrumentation, industrial manufacture, and remote sensing. We have already carried out the proof of concept in this work, with the goal of practicality, and we will consider more practical applications of this new metrological principle on optical measurement and sensing, as well as further engineering improvements to the system in the subsequent research. The performance and compactness might be further enhanced by using the all-fiber architecture to build the entire measurement system and constructing the full-duplex mode in a similar communication system to receive the signal light at the remote end with the fiber that transmits probe light.

## 4. Materials and Methods

### 4.1. Generating Cylindrical Vector Beam

A Sagnac interferometer configuration is used to generate cylindrical vector beam in our experiment, as shown in the generation part of Figure 2(a). A 45° polarized fundamental Gaussian beam is split into an *x* polarized beam and *y* polarized beam by the polarization beam splitter (PBS). The two beams pass through the loop clockwise and counterclockwise. The SPP inserted in the loop converts the fundamental Gaussian beams into optical vortices. Note that the modulated phase twisted chirality is reversed as light passes through the SPP from the front and back, and the twisted phase also turns upside down when reflected by a mirror. After a QWP at 45°, the superposed light field can be expressed as

(6) $$\begin{array}{c} E\left(r,\phi \right)=A_{0}^{\prime }\left(r\right)\cdot \left[\begin{array}{c} 1\\ i\sigma \end{array}\right]\exp\left[i\left(\ell \phi -\beta \right)\right]+A_{0}^{\prime }\left(r\right)\cdot \left[\begin{array}{c} 1\\ -i\sigma \end{array}\right]\exp\left[i\left(-\ell \phi \right)\right], \end{array}$$

where *A*_0_′(*r*) is the real amplitude of optical vortex, *σ* takes ±1 to represent the two circular polarizations, and *β* corresponds to the phase difference of two optical vortices. Obviously, this formula can be derived to Equation (1), with *β* being twice of *α*. As a result, the superposed beam becomes a cylindrical vectorial polarization field after passing through the QWP. In addition, some other approaches for generating vector beams, such as specially designed holograms [52, 53], q-plate [54–56], and fiber-based elements [57, 58], can further simplify our experimental setup, making it closer to the needs of practical applications.

### 4.2. Air-Core Optical Fiber

**Figure 6:**
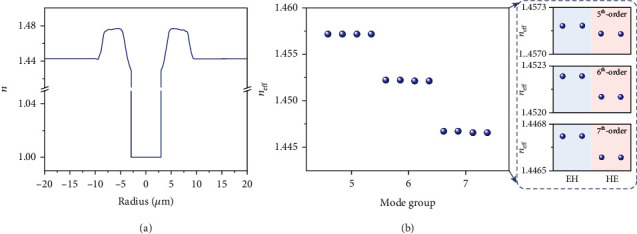
Measured and simulated parameters about the air-core fiber. (a) Measured refractive index profile of the fabricated air-core fiber. (b) Simulated refractive index of mode group 5^th^ to 7^th^.

The manufacturing process and parameters of the air-core fiber have been reported in [48].  Figure 6(a) illustrates the measured refractive index profile of the fabricated air-core fiber, which is plotted with respect to the refractive index of silica. Different from weakly guiding optical fibers, the air-core fiber can support higher-order vector beams transmission, especially for orders equal to 5, 6, and 7. Moreover, losses for mode groups 5^th^ and 6^th^ are measured as 1.9 and 2.2 dB/km, respectively, ensuring that a one-kilometer transmission will not bring a serious loss for vector modes. As shown in Figure 6(b), the refractive index difference of mode groups 5^th^-7^th^ is always higher than 5.8 × 10^−3^. Such a large Δ*n*_eff_ will lead to a large difference of longitudinal wave vector of modes, known as *k*_*z*_ = 2*π* · *n*_eff_/*λ*, keeping the transmitting modes from coupling to other mode groups. In circular fibers, the HE^e,o^ (even and odd) modes are degenerate with each other as the vector fields are related by a rotation. Similarly, EH^e,o^ modes are also degenerate. However, the pairs are not degenerate with each other, *k*_*z*,EH_ ≠ *k*_*z*,HE_, as shown in the zoom-in insets of Figure 6(b). Hence, within the same mode group, most intracrosstalk occurs between even and odd modes under the interference of environment. That is, it is hard for HE modes to couple with EH modes, determining the accuracy of measuring the rotating speed of targets.

### 4.3. Mimicking the Rotating Targets

We employ a high-speed DMD (Texas Instrument DLP7000) to mimic the rotating microparticle. This model of DMD is made up of an array of 1024 × 768 square mirrors with 13.7 *μ*m long. In operation, each micromirror can be switched independently between two tilted states (“ON” and “OFF”), and more precisely, the angles of inclination corresponding to these two states are +12° and −12° with respect to the surface normal, respectively. Considering that each micromirror is switched around an oblique diagonal axis, the DMD panel is mounted on a custom fixture that enables it to revolve 45° to reflect light along horizontal. In the experiment, the rotating target is mimicked by a lump of 78 adjacent micromirrors in the “ON” state, reflecting the signal light back into the optical path for detection, while the other stray lights reflected by the “OFF” state are blocked. This means that the signal light is received along the 12° direction when the probe light illuminates the DMD normally. The angular velocities for the mimicked microparticle are controlled by changing the time interval to continuously switch the DMD patterns prepared beforehand.

## Data Availability

Data underlying the results presented in this paper are not publicly available at this time but may be obtained from the authors upon reasonable request.

## Supplementary Materials

Supplementary Materials Fig. S1: schematic diagram of superposition relationship and intensity, phase, and polarization of fiber eigenmodes in air-core fiber. Fig. S2: (a) schematic of air-core fiber being stressed to form an in-plane bent. (b) Schematic of air-core fiber being waggled to form an out-plane movement. Fig. S3: reconstruction of the polarization distribution of vector beams. (a)–(f) Recorded intensity profiles for calculating the polarization distribution of HE_61_ after transmission: I_*x*_, I_*y*_, I_45_°, I_−45_°, I_RCP_, and I_LCP_, respectively, (g) polarization distribution of HE_61_ after transmission. Table S1: characterized polarization purity of vector beams before and after 1 km air-core fiber transmission. Fig. S4: characterizing the polarization purity of HE_61_ mode after 1 km air-core fiber transmission. Fig. S5: measured results for the rotating target based on the vectorial eigenmodes HE_81_ and EH_61_ in air-core fiber.
