# Supplementary material for: Remote Measurement of the Angular Velocity Vector Based on Vectorial Doppler Effect Using Air-Core Optical Fiber
Source: Research (Wash D C). 2022 Sep 2;2022:9839502. doi: 10.34133/2022/9839502 (PMC9470205; doi:10.34133/2022/9839502)
Supplement: Supplementary Materials — Fig. S1: schematic diagram of superposition relationship and intensity, phase, and polarization of fiber eigenmodes in air-core fiber. Fig. S2: (a) schematic of air-core fiber being stressed to form an in-plane bent. (b) Schematic of air-core fiber being waggled to form an out-plane movement. Fig. S3: reconstruction of the polarization distribution of vector beams. (a)–(f) Recorded intensity profiles for calculating the polarization distribution of HE61 after transmission: Ix, Iy, I45°, I−45°, IRCP, and ILCP, respectively, (g) polarization distribution of HE61 after transmission. Table S1: characterized polarization purity of vector beams before and after 1 km air-core fiber transmission. Fig. S4: characterizing the polarization purity of HE61 mode after 1 km air-core fiber transmission. Fig. S5: measured results for the rotating target based on the vectorial eigenmodes HE81 and EH61 in air-core fiber. [file 9839502.f1.docx]

**Supplementary Information for**

**Remote measurement of the angular velocity vector based on vectorial Doppler effect using air-core optical fiber**

Zhenyu Wan^1,2+^, Yize Liang^1,2+^, Xi Zhang^1,2^, Ziyi Tang^1,2^, Liang Fang^1,2^, Zelin Ma^3^, Siddharth Ramachandran^3^, and Jian Wang^1,2*^

^1^ Wuhan National Laboratory for Optoelectronics and School of Optical and Electronic Information, Huazhong University of Science and Technology, Wuhan 430074, Hubei, China

^2^ Optics Valley Laboratory, Wuhan 430074, Hubei, China

^3^ Boston University, 8 St. Mary’s St., Boston, 02215, USA

+ These authors contributed equally to this work.

* Correspondence to: [jwang@hust.edu.cn](mailto:jwang@hust.edu.cn)

1. Higher-order Modes in Air-core Fiber

Different from weakly guiding optical fibers, the air-core fiber can support both higher-order vector modes and OAM modes transmission, which are described as true fiber eigenmodes. The eigenmodes in optical fiber form a set of orthogonal bases, and the different types of modes can be converted to each other by orthogonal transformation. Typically, the vector modes (HE/EH modes) can be converted by a linear superposition of circularly polarized vortex modes (OAM modes). Fig. S1 illustrates the superposition relationship and intensity, phase, and polarization patterns of fiber eigenmodes with azimuthal index equating to 5 in air-core fiber, namely the EH_4,1_ or HE_6,1_ vector mode group (top row), and the OAM_±5,1_ mode group (middle and bottom row). The solid and dashed lines connecting modes of one group with another represent the corresponding linear combinations, written by

, (S1)

, (S2)

, (S3)

, (S4)

where *σ* takes value 1 for left-circular polarization (LCP) while -1 for right-circular polarization (RCP), *ℓ* is the absolute value of the topological charge of OAM mode, and *i* means there is an additional *π*/2 phase difference in the superposition. The *HE_ℓ_*_+1,1_ mode is composed of vortex modes with a total angular momentum of ±(*ℓ*+1), and the *EH_ℓ_*_−1,1_ mode is composed of vortex modes with a total angular momentum of ±(*ℓ*−1), where ± denotes two kinds of handedness of polarization or OAM.

**
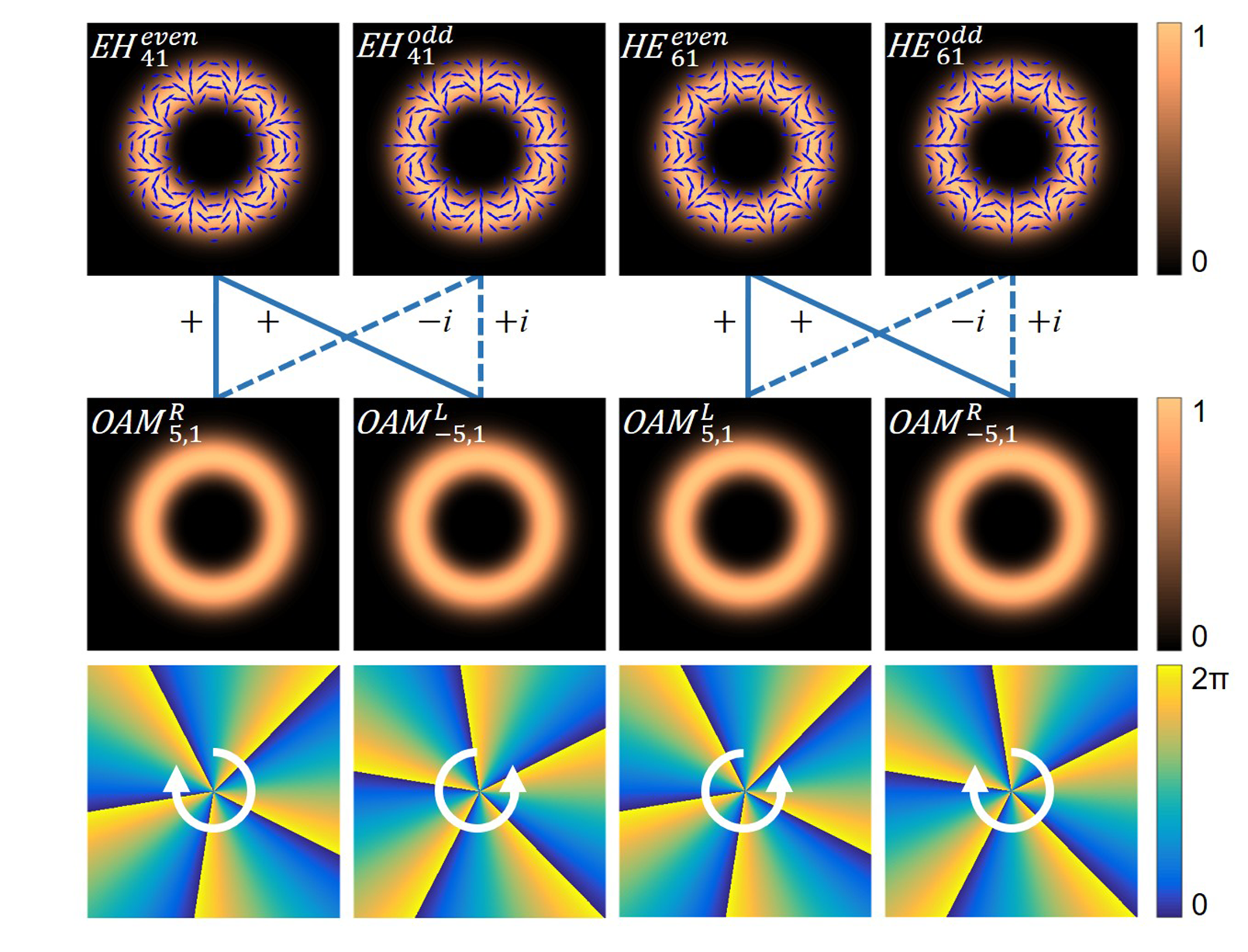
**

**Fig. S1** Schematic diagram of superposition relationship and intensity, phase, and polarization of fiber eigenmodes in air-core fiber. The top row, the intensity and polarization patterns of the vector modes (odd and even modes of HE/EH). The solid and dashed lines show linear combinations modes between groups. The middle row, the intensity patterns of the OAM modes. The bottom row, the phase patterns and polarization of the corresponding OAM modes (clockwise for right-circular polarization and counterclockwise for left-circular polarization).

1. Perturbations in Air-core Fiber

In the Article, two different kinds of perturbations are introduced to the applied air-core fiber. One is stressing the air-core fiber, and the other is waggling the air-core fiber. As displayed in Fig. S2(a), the air-core fiber loop (radius~10cm) is stressed to form a so-called in-plane bent, which corresponds to the stressing situation in the main text. Illustrated in Fig. S2(b) is the schematic of the waggling situation mentioned in the main text, in which the air-core fiber loop is waggled to move from the initial plane (lighter colored loop) to the plane perpendicular to the initial one (darker colored loop) and then moved back. The two kinds of perturbations will lead to different changes of vector modes in air-core fiber, causing the phenomena in Fig. 5 in the main text. The brief analysis is shown below, and details can be found in Ref. [S1].

For the optical fiber with in-plane bent introduced by stressing, birefringence may occur to cause the mixing and crosstalk of degenerate states. However, the OAM mode stability in air-core fiber was already demonstrated [S2], suggesting that OAM modes with high topological charges in air-core fiber are not degenerate states. Given that the birefringence will not lead to different paths of OAM modes with the same total angular momenta within a specific OAM mode group, a vector mode, which is a linear combination of two OAM modes with the same total angular momenta and opposite topological charges, will also possess similar stability.

As to the out-plane movement, an extra geometric phase is added to the transmitted higher-order OAM modes. That is, when the geometric perturbation of the air-core fiber occurs, the light path will attach a solid angle Ω through *k*-space. As a result, the OAM modes will get a change of the so-called Pancharatnam phase proportional to the solid angle, with sign dependents on its topological charge and polarization [S3-S5]. The perturbed higher-order OAM modes (*ℓ* > *σ*) undergoing the geometric phase can be expressed as

, (S5)

where Φ*_g_* is the geometric phase caused by out-plane perturbation, and it takes an opposite sign of the total angular momentum, as shown in equation (2) of Ref. [S5]. As a linear combination of perturbed OAM modes, the perturbed vector modes can be expressed as

, (S6)

, (S7)

, (S8)

. (S9)

Obviously, when the geometric perturbation appears in the fiber, the perturbed vector mode will get partially projected into its degenerate counterpart, that is, crosstalk occurs between the odd and even modes in the mode group. Intuitively, the polarization distribution of the perturbed vector mode rotates somewhat around the central axis. It is remarkable that the crosstalk of perturbed vector mode will not occur between the mode groups, making the air-core fiber greatly robust in our measurement system. By derivation, the electric field of the perturbed vector mode can be described using Jones vector as

, (S10)

where *ϕ* is the azimuthal coordinate, *A*_0_(*r*) is the real amplitude of electric field, Φ_0_ can take 0° and 90° respectively corresponding to odd and even modes, and *σ* takes two values of +1 and -1 respectively corresponding to the *HE_ℓ_*_+1,1_ and *EH_ℓ_*_-1,1_ vector modes. Define *α* = Φ_g_ – Φ_0_, and Eq. (1) in the main text can be obtained as soon as it is. Thus, geometric perturbations, such as those encountered when the fiber is waggled, will change phase factor *α* of the perturbed vector mode.


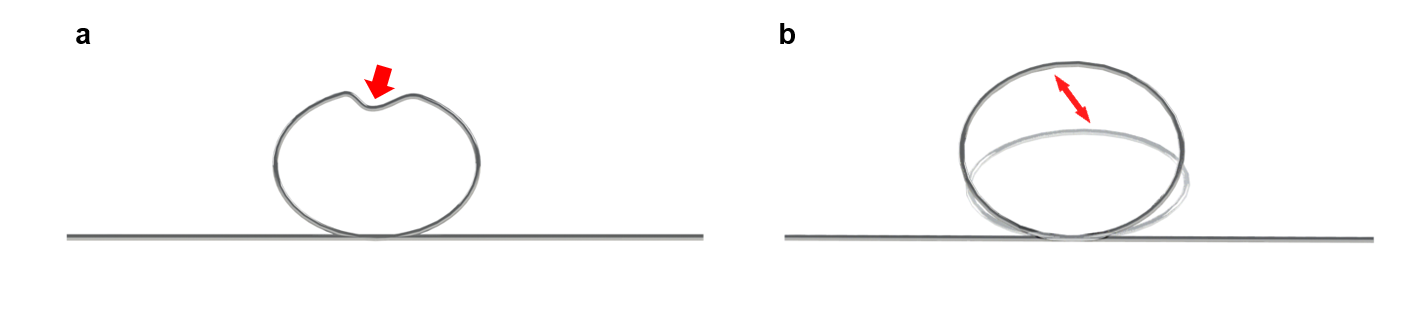


**Fig. S2** (a) Schematic of air-core fiber being stressed to form an in-plane bent. (b) Schematic of air-core fiber being waggled to form an out-plane movement.

1. Reconstruction of the Polarization Distribution of Vector Beams

To make sure that we have produced and transmitted the vector beams, we measure, calculate and then give the polarization reconstruction of the generated vector beams and air-core fiber output beams. The Stokes vector as an expression describing the polarization of a beam can be written as,

$\left\{\begin{matrix} S_{0}=I_{x}+I_{y} \\ S_{1}=I_{x}-I_{y} \\ S_{2}=I_{45^{\circ}}-I_{-45^{\circ}} \\ S_{3}=I_{RCP}-I_{LCP} \end{matrix}\right.$, (S11)

where *I_x_*, *I_y_*, *I*_45°_, and *I*_−45°_ correspond to intensity profiles of beam after passing through an *x*, *y*, 45°, and −45° polarizer, respectively, and *I_RCP_* and *I_LCP_* are the projection of RCP and LCP, measured by passing the beam through a quarter-wave plate (QWP, fast axis: +45° to the *x*-axis) and *x*, *y* polarizer, respectively. Thus, the polarization distribution can be measured and calculated by recording a series of intensity profiles after special polarization devices. The process of reconstructing the polarization distribution of HE_61_ after transmission is displayed in Fig. S3. In our experiment, we firstly insert a polarizer in front of the CCD, and we rotate the angle of polarizer to record the intensity profiles of beam immediately, and then we can get intensity profiles of *I_x_*, *I_y_*, *I*_45°_, and *I*_−45°_, respectively, as shown in Figs. S3a-S3d. Next, we insert a QWP in front of the polarizer. The fast axis of the QWP is adjusted as 45° to the *x*-axis. Rotating the polarizer to *x*-direction and *y*-direction respectively, we finally achieve the record of *I_RCP_* and *I_LCP_*, as shown in Figs. S3e-S3f. According to Eq. (S11), the polarization distribution given by Stokes vectors can be calculated, as shown in Fig. S3g.

**
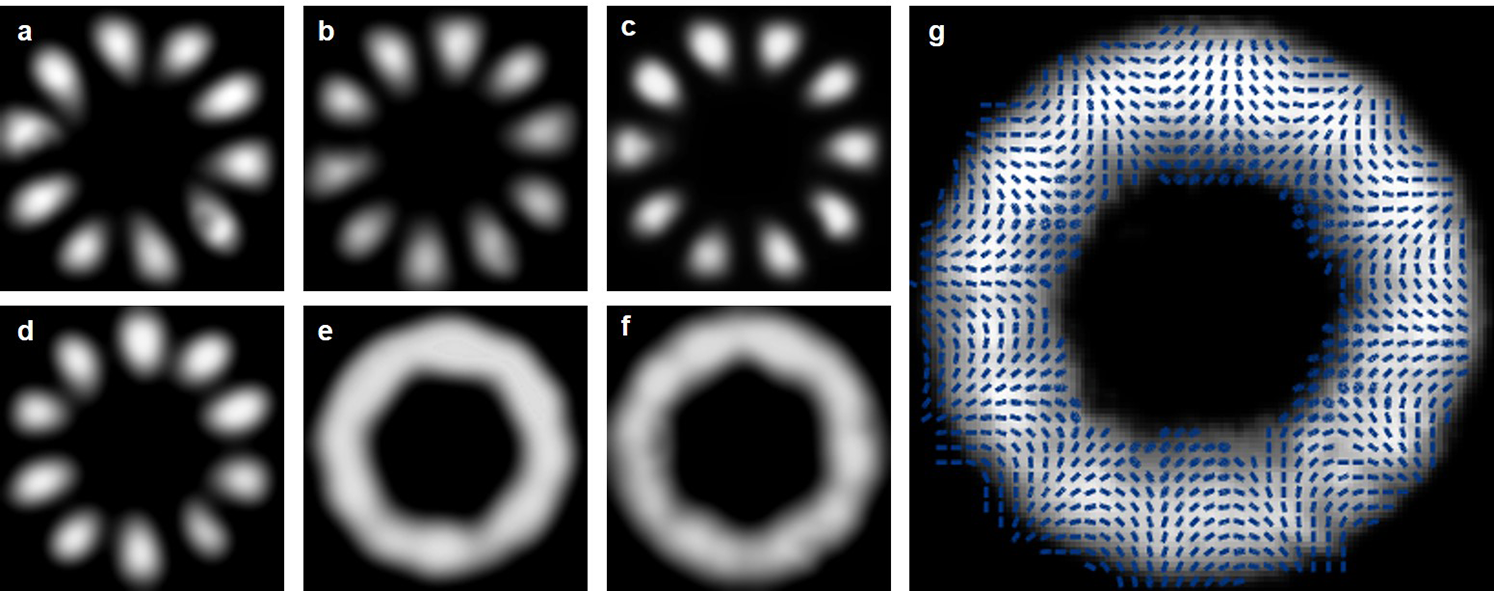
**

**Fig. S3** Reconstruction of the polarization distribution of vector beams. (a)-(f) recorded intensity profiles for calculating the polarization distribution of HE_61_ after transmission: *I_x_*, *I_y_*, *I*_45°_, *I*_−45°_, *I_RCP_*, *I_LCP_*, respectively, (g) polarization distribution of HE_61_ after transmission.

1. Measurement of Polarization Purity of Vector Beams before/after Air-Core Fiber Transmission

The polarization purity of several typically generated vector beams in the main text is quantitatively characterized by calculating the ratio of power with expected polarization to the total beam power, applying the method mentioned in Refs. [S6-S8]. The measured polarization purity of vector beams before and after 1-km air-core fiber transmission is shown in Table S1.

**Table S1.** Characterized polarization purity of vector beams before and after 1-km air-core fiber transmission.

|  | Mode Group 7 | | Mode Group 5 | |
| --- | --- | --- | --- | --- |
| Mode  Place | HE_81_ | EH_61_ | HE_61_ | EH_41_ |
| Before fiber | 90.2% | 91.7% | 91.4% | 92.2% |
| After fiber | 89.0% | 90.1% | 89.7% | 89.5% |

The process of measuring the polarization purity is as follows, taking HE_61_ mode after fiber transmission as an example. The first step is to record six projections of HE_61_ mode, *I_x_*, *I_y_*, *I*_45°_, *I*_−45°_, *I_RCP_*, *I_LCP_*, for determining its Stokes parameters (S0, S1, S2, S3) of each pixel by Eq. (S11), as shown in Figs. S3 (a)-(f). Then, with the calculated Stokes parameters, the direction of the main axis of the local polarization ellipse and the local ellipticity of each pixel can be obtained as $\Psi=1/2\arctan(S_{2}/S_{1})$ and $\chi=1/2\arcsin(S_{3}/S_{0})$, respectively. Fig. S4 (a) illustrates the calculated $\Psi$ distribution of HE_61_ mode after 1-km air-core fiber transmission, and Fig. S4 (b) displays the ideally expected $\Psi_{0}$ distribution of the pure vector beam. For a pure vector mode, which is consist of points with diverse linear polarizations, it features a theoretical polarization distribution , where represents the mode order, is the azimuthal angle and $\theta$ represents a rotation of polarization distribution. To calculate the ratio of power with expected polarization to the total beam power, two parameters are requested, i.e., the angle between measured main axis direction of polarization ellipse and expected polarization direction, and the local ellipticity as well. The angle between measured main axis direction and expected polarization direction can be defined as

$\Delta\Psi=\left|\Psi_{0}-\Psi\right|$, (S12)

which is shown in Fig. S4 (c). And then the polarization purity, which is determined by the ratio of power with expected polarization to the total beam power, can be calculated using

$\eta=\frac{\sum_{i=1}^{m} \sum_{j=1}^{n} I_{0}(i,j)\cos^{2}(\Delta\Psi(i,j))\cos^{2}(\chi(i,j))}{\sum_{i=1}^{m} \sum_{j=1}^{n} I_{0}(i,j)}$, (S13)

where $\eta$ denotes the polarization purity, *m* and *n* represent the pixel number in row and in column respectively, $I_{0}$ corresponds to the intensity profile of vector beam. The molecular represents power with expected polarization, while the denominator denotes the total power of beam.

**
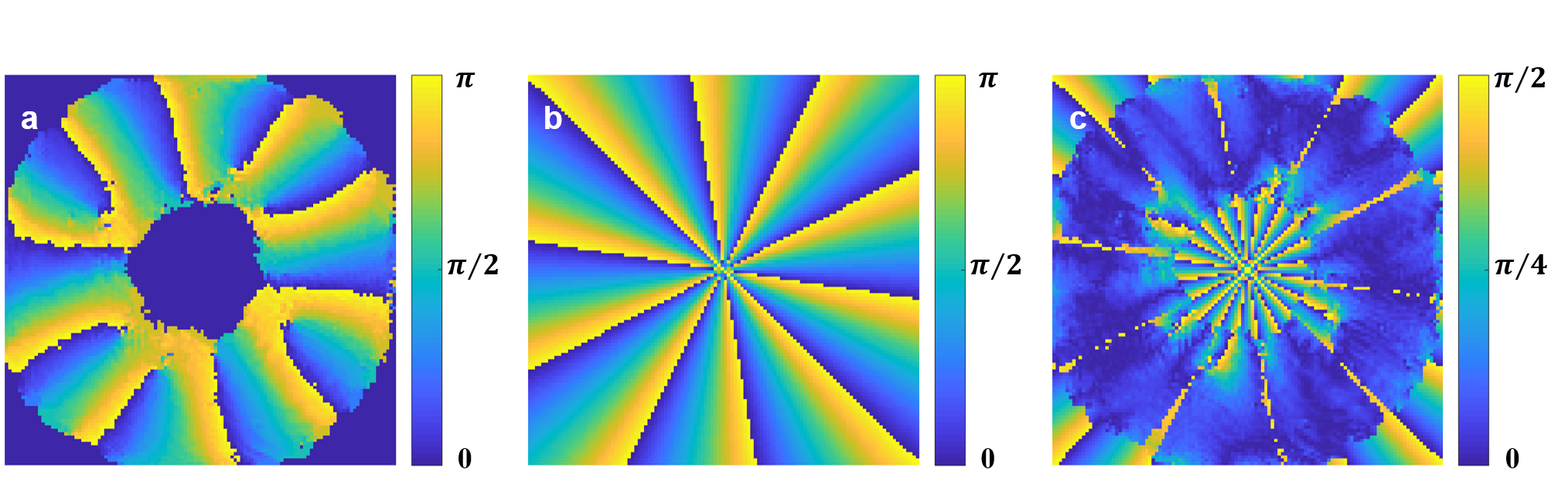
**

**Fig. S4** Characterizing the polarization purity of HE_61_ mode after 1-km air-core fiber transmission. (a) Calculated angle of the main axis of the local polarization ellipse: Ψ wrapped to 0-π; (b) Expected polarization angle of each pixel of pure vector beam: Ψ_0_ wrapped to 0-π; (c) Calculated angle between measured main axis direction and expected polarization direction: ΔΨ wrapped to 0-π/2.

1. Additional Results for Measuring the Angular Velocity Vector

Beyond the measured results shown in Fig. 3 of the main text, here we present more experimental results for the rotating target. Shown in Fig. S4 are detected Doppler intensity signals (Figs. S4a, S4d, S4g, S4j), calculated amplitude spectra of Fourier transformation (Figs. S4b, S4e, S4h, S4k), and relative phase difference spectra between two intensity signals (Figs. S4c, S4f, S4i, S4l) based on the vectorial eigenmode HE_81_ and EH_61_ in air-core fiber. Via HE_81_ mode, we acquire a set of data as the mimicked microparticle rotating counterclockwise with Ω = 20π rad/s (Figs. S4a-S4c) and clockwise with Ω = −20π rad/s (Figs. S4d-S4f), respectively. Likewise, via EH_61_ mode, we acquire another set of data (Figs. S4g-S4l). Obviously, when a fixed vector mode is used as the probe light, the two intensity signals reveal a significant lead or lag relationship in different rotation directions, and the corresponding relative phase difference matching the frequency peak is also reversed. From the comparison, even if the vector mode of the probe light is replaced, the results obtained under different rotation directions can still be distinguished by the inversion of the relative phase difference. The additional results also further demonstrate the successful implementation of full vector information extraction of the angular velocity, i.e., the measurement system is applicable under different vector modes.


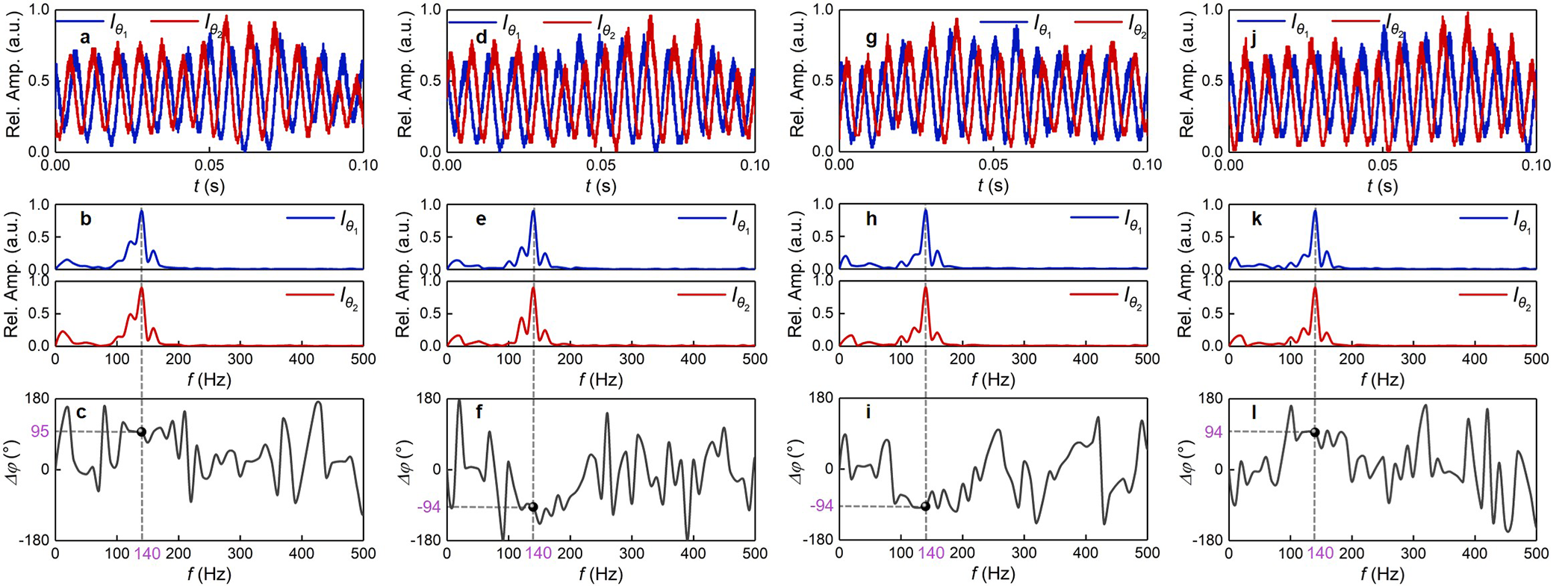


**Fig. S5** Measured results for the rotating target based on the vectorial eigenmode HE_81_ and EH_61_ in air-core fiber. (a)-(c), (g)-(i) measured results for counterclockwise motion with Ω = 20*π* rad/s. (d)-(f), (j)-(l) measured results for clockwise motion with Ω = −20*π* rad/s. (a)-(f) measured results via HE_81_. **(**g)-(l) measured results via EH_61_. (a), (d), (g), (j) measured Doppler intensity signals by PD1 and PD2 after filtering the DPS through two polarizers, respectively. (b), (e), (h), (k) calculated Fourier amplitude spectra for obtaining a Doppler frequency shift peak. (c), (f), (i), (l) calculated relative Fourier phase spectra between two intensity signals for obtaining the relative phase difference matching the frequency peak.

**References**

1. Z. Ma, and S. Ramachandran, "Propagation stability in optical fibers: role of path memory and angular momentum." *Nanophotonics* **10**(1), 209-224 (2021).
2. P. Gregg, P. Kristensen, and S. Ramachandran, “Conservation of orbital angular momentum in air-core optical fibers,” *Optica* **2**(3), 267-270 (2015).
3. A. Tomita and R. Y. Chiao, “Observation of Berry's topological phase by use of an optical fiber,” *Phys. Rev. Lett.* **57**(8), 937-940 (1986).
4. E. J. Galvez et al., “Geometric phase associated with mode transformations of optical beams bearing orbital angular momentum,” *Phys. Rev. Lett.* **90**(20), 203901 (2003).
5. G. Milione et al., “Higher order Pancharatnam-Berry phase and the angular momentum of light,” *Phys. Rev. Lett.* **108**(19), 190401 (2012).
6. Z. Bomzon, V. Kleiner, and E. Hasman, “Formation of radially and azimuthally polarized light using space-variant subwavelength metal stripe gratings,” *Appl. Phys. Lett.* **79**(11), 1587-1589 (2001).
7. G. Machavariani et al., “Birefringence-induced bifocusing for selection of radially or azimuthally polarized laser modes,” *Appl. Opt.* **46**(16), 3304-3310 (2007).
8. Y. Zhang et al., “Controllable laser output of high-quality cylindrical vector beam through intra-cavity mode conversion,” *Appl. Phys. Lett.* **117**(11), 111105 (2020).
